# Perforation of small intestine as a rare adverse event of acupuncture: a case report

**DOI:** 10.1186/s12245-026-01164-7

**Published:** 2026-03-16

**Authors:** Xiangyun Zheng, Wenchang Wang, Shuangshuang Zhang, Shaowei Sun, Huanhu Zhang

**Affiliations:** 1 Department of Gastrointestinal Surgery, Cheeloo College of Medicine, Weihai Municipal Hospital, Shandong University, 70 Peace Road, Weihai, Shandong Province 264200 China; 2 Department of Experimental Training Center, Shandong Drug and Food Vocational College, Weihai, Shandong Province 264210 China

**Keywords:** Acupuncture, Adverse events, Small intestine, Intra-abdominal infection, Intestinal obstruction

## Abstract

**Background:**

With the widespread application of acupuncture, there is growing concern about the adverse events (AEs) related to therapy. Acupuncture can result in severe life-threatening AEs in very rare cases, so there are important clinical implications of identifying and reporting the cause of AEs to prevent similar incidents in the future. Herein, we report a rare life-threatening case of small intestine perforation caused by acupuncture.

**Case presentation:**

A 71-year-old man was transferred to our emergency department for further treatment due to intestine obstruction with a giant abdominal wall hernia. Before admitting to our hospital, the patient had previously been treated with acupuncture and the acupoints were located on the abdomen and limbs. In view of progressively worsening abdominal pain and intra-abdominal infection, he underwent an emergency exploratory laparotomy. Intraoperative findings confirmed that four perforations of the small intestine were caused by acupuncture.

**Conclusion:**

This case report demonstrates that abdominal acupuncture in patients with concurrent intestinal obstruction and a giant abdominal wall hernia carries an extreme risk of iatrogenic bowel perforation, due to the loss of normal anatomical safeguards. Therefore, in this specific high-risk scenario, abdominal needling should be approached with extreme caution or considered contraindicated. Meticulous pre-treatment anatomical assessment is imperative to prevent this life-threatening complication.

## Background

Acupuncture, a widely used alternative and complementary therapy, has gained increasing attention worldwide in the past decades. As an effective treatment modality, acupuncture mainly stimulates acupoints along the meridian channels to treat a variety of medical conditions and symptoms [1]. With the widespread application of acupuncture, there is growing concern about the adverse events (AEs) related to therapy [1–4]. The incidence of acupuncture-related AEs has been reported to be 3.76–8.6% [5]. The most common AEs were minor bleeding and needling pain, while in very rare cases, acupuncture can lead to severe life-threatening AEs, such as pneumothorax [5], cardiac perforation [6], and cardiac tamponade [7]. Therefore, there are important clinical implications of identifying and reporting the cause of AEs to prevent similar incidents in the future. So far, there are several reported cases about the acupuncture-related organ injury, such as the heart, lungs, spinal cord, brain, eye and liver, but small bowel perforation has rarely been reported [1, 3, 8, 9].

## Case presentation

**Fig. 1:**
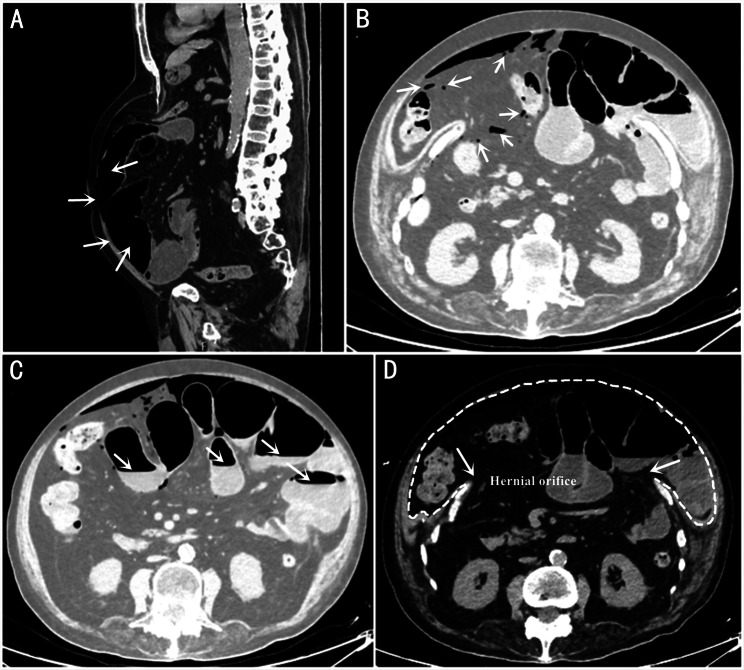
Imaging findings on contrast-enhanced CT. (**A**) the sagittal position showed dilated intestinal lumen, extensive gas within the intestinal lumen, and an abdominal wall fascial defect in the upper quadrant, (**B**) large amount of free gas visible in the abdominal cavity, (**C**) multiple air-fluid levels in the small bowel, (**D**) CT of the upper abdomen (axial view) showed an abdominal wall fascial defect, approximately 15 cm in the greatest diameter, and the small intestine was herniated into the subcutaneous layer to form a giant abdominal wall hernia, white arrowheads indicated the defect in the abdominal wall musculature and fascia (hernial orifice), the contour of the hernia sac was outlined by a white dashed line

A 71-year-old man, who was initially diagnosed with intestinal obstruction at an outside hospital, was transferred to our emergency department presenting with abdominal pain, abdominal distention, and obstipation for 8 days. He had a past medical history of ankylosing spondylitis for more than 20 years, for which he reported a history of oral NSAID use but had not received biologic therapy, a history of abdominal wall hernia for 10 years which had remained untreated, and no history of surgery or other diseases such as peptic ulcer disease. Before admitting to our hospital, the patient had previously been treated with acupuncture for intestinal obstruction by a licensed practitioner at a local clinic, but had shown no obvious improvement. Abdominal examination revealed marked abdominal distension, diffuse abdominal tenderness without rebound tenderness, and hypoactive bowel sounds. The results of laboratory examination were as follows: white blood cells count 6.45 × 10^9^/L(reference range: 3.5–9.5 × 10^9^/L), neutrophil-to-lymphocyte ratio 89.2%(reference range: 40–75%), red blood cells count 4.72 × 10^12^/L(reference range: 4.3–5.8 × 10^12^/L), Hb 150 g/L(reference range: 130–175 g/L), platelet count 181 × 10^9^/L(reference range: 125–350 × 10^9^/L), C-reactive protein 112.78 mg/L(reference range:<3.0 mg/L), procalcitonin 0.72 ng/mL(reference range: 0-0.06ng/mL), normal renal and hepatic values, normal pH, lactate and base excess. Emergent contrast-enhanced computed tomography (CT) scan was performed to further evaluate the cause and severity of the intestinal obstruction upon admission. Abdominal CT demonstrated dilated bowel loops(Fig. 1A and C), extensive gas within the intestinal lumen (Fig. 1A and C), massive intra-abdominal free gas (Fig. 1B), multiple air-fluid levels in the small bowel (Fig. 1C), a giant abdominal wall hernia with a defect measuring approximately 15 cm in greatest diameter, and the small intestine was herniated into the subcutaneous layer to form a giant abdominal wall hernia (Fig. 1A and D). In addition, there were no signs of intra-abdominal tumor, intestinal torsion, internal abdominal hernia and mesenteric infarction. The presence of massive intra-abdominal free gas suggested alimentary tract perforation. Considering the history of receiving acupuncture treatment, we urgently revisited the details of the procedure with the patient. The acupuncture was confirmed to be manual needling utilizing sterile, single-use silver needles (length 25 mm, diameter 0.30 mm). The acupoints were located on the abdomen and limbs, with the abdominal points primarily including Tianshu (ST25), Shuidao (ST28), and Zhongwan (RN12). Critically, some needles had been inserted in the upper abdominal region, which corresponded precisely to the area of the pre-existing giant abdominal wall hernia. Each session lasted approximately 30 min, and the treatment had been administered once daily for two consecutive days prior to transfer. The patient reported that none of the stuck needles, broken needles, bent needles, or embedded needle migrations occurred during the process.

Based on the imaging findings on contrast-enhanced CT, abdominal acupuncture, and a giant abdominal wall hernia, we suspected that the patient had experienced perforations of the small intestine, which were caused by acupuncture. We therefore considered that the patient currently had surgical indications and recommended immediate surgical intervention. Although the CT scan revealed pneumoperitoneum, indicative of perforation, the patient’s abdominal pain was initially mild and localized, without signs of generalized peritonitis (e.g., board-like rigidity, severe rebound tenderness), the patient initially opted for conservative management. On the next morning after admission, he developed significant symptoms of fever, abdominal muscle tension, rebound pain, and worsening abdominal pain and distention, which necessitated an emergency exploratory laparotomy Intraoperative exploration revealed that there were four perforations of the small intestine, which were located in the jejunum (140 cm, 150 cm, and 190 cm from the ligament of Treitz) and ileum (120 cm proximal to the ileocecal junction), respectively. Each of the perforations had a diameter of approximately 5 mm, which was covered with purulent exudates. The 100 cm of ileum immediately anterior to the ileo-cecal junction showed extensive dense adhesions. The proximal small intestine was significantly dilated, congested, and oedematous, while the distal colon and rectum were collapsed. Furthermore, the giant abdominal wall hernia was confirmed and there was no perforation of the stomach, duodenum, colon, rectum, or gallbladder. After copious warm saline wash-out, enteric decompression was performed via the ileal perforation site, then all the perforations were sutured and an extensive bowel adhesiolysis was performed. Due to the existence of intra-abdominal contamination and infection from intestinal perforation, definitive tension-free abdominal wall hernia repair (which typically involves synthetic mesh) was not performed. This decision adhered to the fundamental surgical principle of avoiding permanent prosthetic material in a contaminated field, due to the high risk of mesh infection and subsequent surgical failure. The hernia defect was left for potential delayed repair in a subsequent, elective procedure after complete resolution of the infection. Postoperative recovery was uneventful with good incision healing, and the patient was discharged on the 11th postoperative day.

## Discussion

Given the absence of prior abdominal surgery, the giant abdominal wall hernia in this case represents a primary ventral hernia. The etiology of primary ventral hernias is often multifactorial, involving chronic increases in intra-abdominal pressure and weakening of the abdominal wall musculature or fascia [10]. In this patient, potential contributing factors over the 10-year course could include chronic, unmitigated strain related to his long-standing ankylosing spondylitis, which may have led to altered posture and respiratory mechanics, thereby elevating chronic intra-abdominal pressure [11]. Additionally, inherent connective tissue vulnerability or age-related muscular atrophy might have predisposed him to fascial weakness. According to the intraoperative findings, the cause of the intestinal obstruction was extensive dense adhesion of the ileum. As the patient had no history of abdominal surgery, the causes of adhesion formation were not definitive. Adhesive bowel obstruction, one of the common surgical emergencies, can usually be settled with non-operative management unless the signs of strangulation, intestinal ischemia, or peritonitis are observed [12]. Acupuncture has been confirmed its efficacy in the non-surgical treatment of adhesive bowel obstruction [13, 14]. Tianshu (ST25), Shuidao (ST28), and Zhongwan (RN12) are the commonly used abdominal acupoints [14]. Accumulated evidence has denoted that acupuncture exerts gastrointestinal modulatory effects through neuromodulation and anti-inflammatory pathways [15, 16].

However, the safety of abdominal acupuncture is predicated on normal anatomy. In standard practice, fine needles (such as the 0.30 mm diameter, 25 mm long needles used in this case) are inserted to a moderate depth (typically 10–25 mm) at points like ST25 and RN12, intended to stimulate subcutaneous and muscular layers without breaching the peritoneum. This safety margin is critically compromised in the setting of a giant abdominal wall hernia. The hernia sac, comprising only attenuated skin, subcutaneous tissue, and peritoneum, lacks the robust muscular and fascial barrier of a normal abdominal wall. Concurrently, the obstructed and markedly dilated small intestine within the hernia is pushed directly against this thin tissue layer. This combination—a thinned, defenseless abdominal wall overlying a tense, distended bowel loop—drastically reduces or eliminates the effective tissue buffer. Consequently, a needle inserted with a standard depth and technique can easily achieve transperitoneal penetration and directly injure the intestinal wall, which is the direct mechanical etiology of the iatrogenic perforations in this case. The morphology and distribution of the perforations observed during surgery provide strong corroborative evidence for this iatrogenic needle injury mechanism. The four discrete, small (approximately 5 mm in diameter), rounded perforations at different levels of the small intestine are highly characteristic of penetrating trauma from sharp, thin objects like acupuncture needles. In contrast, perforations secondary to pressure necrosis from obstruction or ischemic injury typically present as larger, irregular, and often discolored or necrotic patches of bowel wall, and are usually solitary or clustered within a single compromised segment. While spontaneous perforation can occur in severely dilated bowel, it is rare and typically manifests as a single, larger rent rather than multiple, discrete punctures. Therefore, the operative findings of multiple, small, round holes are inconsistent with the expected presentation of obstruction-related ischemic or pressure necrosis, and are far more congruent with direct mechanical penetration by needles, supporting acupuncture as the direct cause.

Many acupuncture-related AEs have been reported and most of them were due to improper technique [2]. Improper needling techniques including stuck needles, broken needles, bent needles, and embedded needle migrations are generally identified as the leading cause of AEs [2, 3]. None of the above-mentioned situations were present in our case, and the selected abdominal acupoints were classic acupoints that are commonly used for the treatment of bowel obstruction. In identifying the cause of acupuncture-related AEs, patient factors need to be considered, especially the anatomical variations and pathophysiological specificity of individual patients [4]. The giant primary ventral hernia in this case had allowed the small intestine to herniate into the subcutaneous space. Moreover, the small intestine was significantly dilated. Under such circumstances, the acupuncture needle can be easily brought through the abdominal wall and inserted into the intestinal lumen. More importantly, the puncture wounds of the intestinal wall were unable to self-heal in the presence of obstruction and marked intestinal distension. Subsequently, the bowel contents continuously flowed into the abdominal cavity, leading to persistent and worsening intra-abdominal infection. As a result, conservative treatment was not effective, and eventually the surgical intervention could not be avoided. From these, it can be seen that the reasons for the small bowel perforation in our patient were related to the anatomical changes of abdominal acupoints and the pathological alterations of bowel obstruction. Surgical treatment eventually cured the patient through small intestinal decompression and the release of intestinal obstruction.

This case report has several inherent limitations. First, as a single-case report, it highlights a potential life-threatening hazard rather than quantifying its incidence. Second, the retrospective nature limits definitive causal inference, though the sequence of events strongly supports it. Third, the pathophysiological mechanism, while plausible, is derived from this specific scenario. Most importantly, the conclusion is strictly limited to the high-risk combination of intestinal obstruction with a giant abdominal wall hernia and should not be overgeneralized to other patient populations.

## Conclusion

This case demonstrates that abdominal acupuncture in patients with both intestinal obstruction and a giant abdominal wall hernia carries an extreme risk of iatrogenic bowel perforation. The loss of the normal anatomical barrier and the proximity of distended bowel loops fundamentally compromise the standard safety profile of this procedure. Therefore, a meticulous pre-treatment anatomical assessment is imperative. Clinicians must prioritize individual patient anatomy over the routine application of standard acupoint protocols. In this specific high-risk clinical scenario, abdominal acupuncture should be approached with extreme caution or considered contraindicated to prevent life-threatening complications.

## Data Availability

Data sharing is not applicable to this article as no datasets were generated or analysed during the current study.

## Abbreviations

AEs: Adverse events
CT: Computed tomography
